# “Terrifying and Boring”—Reflecting on 30 Years of *Emerging Infectious Diseases*

**DOI:** 10.3201/eid3112.AC3112

**Published:** 2025-12

**Authors:** D. Peter Drotman

**Affiliations:** Editor-in-Chief Emeritus, *Emerging Infectious Diseases*, Atlanta, Georgia, USA

**Keywords:** emerging infectious diseases, art-science connection, COVID-19

**Figure:**
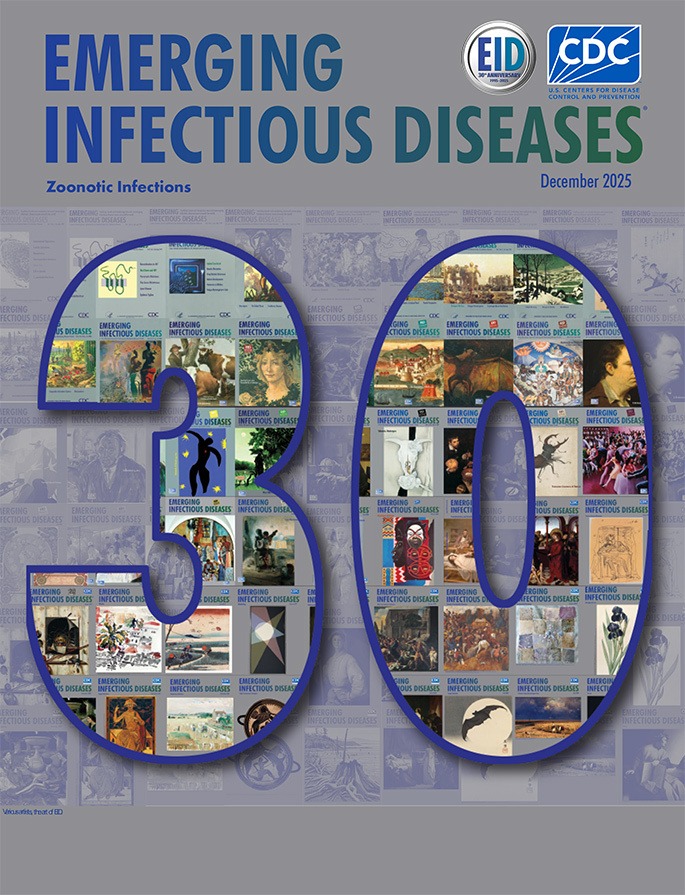
**Compilation of *Emerging Infectious Diseases* covers,** various artists, 2025. Digital design by Reginald Tucker.

“We get these at work—the covers are interesting and look good in the break room, the content is highly specialized and beyond me, and those entries that aren’t too technical for lay reading balance themselves between terrifying and boring. Of course, if you’re an ID doc, there’s probably a different impression drawn.”

—2010 anonymously posted comment on EID’s content

This month’s cover celebrates the 30th anniversary of the publication of *Emerging Infectious Diseases*, the Centers for Disease Control and Prevention’s initial foray into the world of peer-reviewed, open access, biomedical publishing. After 3 decades of continuous publication, including more than 13,000 articles that have accrued hundreds of thousands of citations, the foray has been successful, but that success was not assured from the onset.

The term “emerging infections” was not a familiar one in 1995 when EID’s inaugural issue appeared. The very first article in the first issue was a declaration by CDC director (and later Surgeon General of the United States) David Satcher that “New infectious diseases, often with unknown long-term public health impact, continue to be identified” (*1*).

The world owes a debt of gratitude to the late Dr. Joshua Lederberg (1925–2008) for recognizing and ultimately addressing emerging infections in all their aspects from the molecular to the global. After winning the Nobel Prize in 1958 at the age of 33, Dr. Lederberg embarked on an impressive career that involved becoming a science advisor to many US presidents and, in 1992, leading the Institute of Medicine (IOM) to issue a report that not only described emerging infections but also defined them and recommended a strategy to address them (*2*). Many factors prompted Dr. Lederberg to join with prolific arbovirologist Robert Shope (*3*) to produce that landmark report, which included the HIV/AIDS pandemic, increases in tuberculosis, development of antimicrobial resistance, threats of pandemic influenza, and recognition of viral hemorrhagic fevers, all in the face of diminished public health capacity to address those issues. Part of the resulting report recommended that the United States fund research and training in microbiology, clinical infectious disease, and public health. One of the many actions proposed was the establishment of an outlet for the fruits of the research the IOM envisioned would be carried out. *Emerging Infectious Diseases* owes its genesis to that recommendation. The founding editors declared, “*Emerging Infectious Diseases* was intended less as an archive of science and more as a means of communicating science to the interdisciplinary public health community. The central premise was that global dissemination of emerging disease information was urgent and the reader was extremely busy” (*4*).

When it began in 1995, the journal was a quarterly publication and included many review articles that served to provide the public health community with the information needed to understand what diseases were emerging and how to begin to address them. From the beginning, the journal was open access (free to readers and authors). Articles were kept short and the number of references limited, features generally appreciated by the intended audience of frontline public health practitioners, but not always loved by contributing authors. Examples of such articles from the earliest years of EID include several topics that have been cited thousands of times: biofilms (*5*), food safety (*6*), and antimicrobial resistance (*7*).

The articles that made EID must-reading for the public health and infectious disease communities were ones that brought extraordinary attention to emerging infections in 2001: a special issue on West Nile virus (*8*) and a fast-track report of cases of bioterrorism related inhalational anthrax (*9*). Beginning in 2002, EID became a monthly journal. By 2003, the number of submissions had increased markedly, and the journal transitioned to an online submission and peer review system. Over time, the journal’s submission totals increased to 2,000 articles per year, with some notable exceptions: during 2020, the first year of the COVID-19 pandemic, the journal received nearly 5,000 article submissions and published a substantial number more articles than average to accommodate the surge in research into this critical emerging pathogen.

Consistent leadership has been a benefit during the journal’s publication history; the journal has had only 3 Editors in Chief and 3 Managing Editors (Table). Over the years, the journal has consistently ranked in or near the top 10% of the various categories in which it is assigned by the major citation evaluation institutions. The journal posts such metrics on its About the Journal page on the EID website (https://wwwnc.cdc.gov/EID/about).

**Table:** Tenures of Editors in Chief and Managing Editors for *Emerging Infectious Diseases*, 1995–2025

Title	Name	Years
Editor in Chief	Joseph McDade	1995–2001
	D. Peter Drotman	2001–2025
	Matthew J. Kuehnert	2025–
Managing Editor	Polyxeni Potter	1995–2014
	Byron Breedlove	2014–2025
	Lesli Mitchell	2025–

In March 1997, the journal first featured images on its cover, initially scientific illustrations. In December 1997, EID began showcasing artwork with brief details about the imagery on its covers; explanatory essays to accompany the artwork began in October 2000. The journal’s cover art and images set it apart from many other journals, and the practice was the subject of a 2018 article in the Columbia Journalism Review that the journal “appears to have something of a niche following among art and literary types, who appreciate the magazine for its beautiful covers and dismal content” (*10*). Even though EID discontinued hardcopy printing in 2020, the journal still produces monthly issues with cover images, and they continue to be very popular with the readership and others. The images are intended to humanize the content, recounting, as the articles often do, the ills of people and animals. As those ills have afflicted every human community in every geographic area, every culture, and every period in history, the range of cover images is intended to reflect all genres of art, all artistic disciplines, and all periods of history. The hundreds of cover images are available on the journal website (https://wwwnc.cdc.gov/EID/past-covers). A collection of past covers and essays was also presented in a book by founding Managing Editor Polyxeni Potter in 2013 (*11*).

A few features of EID that have proved serendipitously popular with readers are the Etymologia and Photo Quizzes, both of which have a focus on history. The Etymologia feature began with the August 2005 issue as filler material to provide some content in space that would otherwise be blank. A copyeditor simply inserted the etymology of a word or organism name, but the journal’s editors came to understand its engagement with the EID readership when the journal literally missed the boat by omitting the story behind the true etymology of *Pseudoterranova azarasi* in the March 2011 issue. That Etymologia had merely translated the Greek and Latin words for false and new and land, but knowledgeable readers pointed out that *Terranova* was the name of Antarctic explorer Robert Falcon Scott’s ship and that among the members of the crew were naturalists who documented new species of aquatic creatures and their parasites as they sailed into uncharted southern waters. The story of how they named a species after their ship prompted EID to delve further into the derivation of the terms published.

One way EID highlights the heritage of emerging infections is to present photo quizzes as brief historical essays, the photo being a portrait of a scientist whose contributions deserve to be brought to the attention of the next generation of infectious disease and public health practitioners and investigators. The feature began in 2008 with a portrait of pioneering cellular pathologist Rudolf Virchow and this clue: “For if medicine is really to accomplish its great task, it must intervene in political and social life. It must point out the hindrances that impede the normal social functioning of vital processes, and effect their removal” (*12*).

The continued emergence of new infections, the reemergence of once-controlled infections, and the rapid succession of pandemic infections support the continued need for EID. The goal has never been to compete with the many other fine clinical infectious disease journals, some of which have been in press for more than a century. EID’s prevention and public health orientation means that its scope goes far into the veterinary and environmental spheres. For example, since 2005, EID has devoted each of its December issues to zoonotic and veterinary topics (*13*), in no small part because the world has taken note that a great many emerging infections result from spillover events. That emerging infectious diseases appear periodically, ranging from avian influenza to Zika, suggests that the need to address, control, and chronicle them will be with us for at least another 30 years—and likely for many more decades beyond.
